# SGIS-RC: Bio-Inspired Identifiability-Aware Single-Anchor Scalar-Range Compensation

**DOI:** 10.3390/biomimetics11100692

**Published:** 2026-09-24

**Authors:** Xiang Zhang, Huaixiang Zhang, Ertao Li, Zhirong Wang

**Affiliations:** 1 School of Computer Science, Hangzhou Dianzi University, No. 1158, 2nd Street, Qiantang District, Hangzhou 310018, China; gavin@hdu.edu.cn (X.Z.); tonylee@hdu.edu.cn (E.L.); 2 Changzhou Institute of Inspection and Testing Standard Certification, No. 2-18 Xihu Road, Wujin District, Changzhou 213164, China; zhirongwang@zju.edu.cn

**Keywords:** bio-inspired design, scalar-range compensation, single-anchor sensing, robot calibration, numerical identifiability, support-aware learning

## Abstract

Biological control often combines selective sensing, complementary response scales, and attenuation under weak evidence. Guided by these principles, this paper presents Spectrum-Guided Identifiable Single-Anchor Range Compensation (SGIS-RC), a deterministic physics–data framework for single-anchor scalar-range compensation. SGIS-RC removes the local anchor–bias nuisance tangent from the kinematic sensitivity, retains a numerically conditioned physical subspace, and models the remaining discrepancy through a Chebyshev–Matérn global–local residual field. The learned correction is further regulated by leverage, configuration-space support, and bounded amplitude control. On two public workbooks, ABB IRB120 and HSR-C JR680, a pre-specified outer-partition evaluation performed after the model specification was frozen yielded configuration-group MAEs of 0.2306±0.0148 mm and 0.0493±0.0021 mm, respectively, while the matched conventional hybrid yielded slightly lower mean errors of 0.2244±0.0151 mm and 0.0478±0.0015 mm. Same-fit analyses show that support placement can alter the balance between global and local residual correction, and the block protocols indicate that support-shift behavior remains dataset dependent. Overall, SGIS-RC provides a structured and auditable framework that integrates identifiability-aware physical adaptation, global–local residual modeling, and support-aware deployment control for single-anchor scalar-range compensation.

## 1. Introduction

Industrial robot calibration improves absolute positioning by correcting discrepancies between a nominal model and the realized mechanism. Classical calibration relies on accurate external metrology and sufficiently informative configurations; consequently, estimation quality depends not only on the optimizer but also on measurement geometry, parameterization, and observability [1,2,3,4]. Laser trackers provide high-quality Cartesian references but may be costly or inconvenient for routine in-cell recalibration [5,6]. Distance-based alternatives—including ball-bar, distance-sphere, cable, and range-sensor systems—replace full pose observations with simpler geometric constraints and have therefore attracted sustained interest for economical calibration [7,8,9,10,11].

Bio-inspired engineering offers a complementary way to organize this inference problem. Biological sensory and sensorimotor systems preserve reliable behavior by suppressing task-irrelevant variation, adapting their response to environmental statistics, and weighting or attenuating responses according to the reliability of available evidence [12,13,14]. In robotics, biological inspiration can be used as an abstraction of organizational principles rather than as a literal copy of anatomy [15]. Applied to calibration, this perspective suggests an estimator that selectively retains informative physical directions, learns discrepancy at complementary global and local scales, and limits adaptive correction when a query is weakly supported by prior experience.

The economy of a single range measurement comes with a sharper inferential limitation. For tool position p(q;θ), anchor *a*, and range bias *b*, the observation depends on ${∥p-a∥}_{2}+b$. Over a restricted configuration set, perturbations of θ, *a*, and *b* may generate nearly collinear first-order effects. Directly estimating all kinematic corrections can therefore amplify weak directions and transfer nuisance variation into physically uninterpretable updates. A fixed anchor removes part of this coupling, but it defines a different estimand from deployment in which the anchor itself must be inferred.

A second limitation appears after physical fitting. Compliance, transmission, thermal, and measurement effects generally leave configuration-dependent discrepancies that motivate data-driven compensation. Recent work has advanced this direction through learned error models, Gaussian-process residuals, and data-efficient experiment design [16,17,18,19]. Nature-inspired optimization has also been introduced into robot calibration through diversified evolutionary ensembles and particle-swarm-optimized hybrid networks [20,21]. These developments improve predictive accuracy or measurement efficiency, but they also sharpen a complementary question for scalar-range compensation: how should learned compensation be prevented from absorbing weak physical directions or extrapolating without control beyond the configurations that support the fit?

SGIS-RC addresses these two validity boundaries by translating three principle-level biological abstractions into a deterministic physics–data estimator: selective filtering of nuisance variation, complementary global–local adaptation, and reliability-dependent attenuation of learned correction. The framework does not reproduce a biological organ and does not estimate biological quantities; its bio-inspired character concerns the organization of information processing and adaptive regulation. The individual numerical and statistical tools are established; the contribution lies in their problem-specific organization under the single-anchor scalar-range measurement contract and in the auditable separation of physical, residual, and deployment roles. The contributions are:

(1) a selective, nuisance-projected spectral formulation that restricts single-anchor physical updates to locally separable and numerically supported directions while keeping unknown- and fixed-anchor estimands distinct;
(2) a complementary global–local Chebyshev–Matérn residual model with leverage, support, and amplitude controls, providing an adaptive but explicitly regulated learned contribution as deployment support weakens;
(3) a multi-protocol evaluation combining repeated-partition performance, same-fit mechanism analyses, sensitivity and deployment-control studies, and a separately pre-specified post-specification outer-partition evaluation of the frozen model specification without treating repeated partitions as independent robot replications.

## 2. Related Work

### 2.1. Bio-Inspired Data-Driven Regulation

Bio-inspired robotics transfers organizational principles from living systems into engineering rather than requiring a one-to-one reproduction of biological structure [15]. Three principles are particularly relevant to data-driven calibration. First, sensory adaptation changes effective gain in response to environmental statistics, allowing a system to emphasize informative variation while avoiding indiscriminate amplification [12]. Second, biological perception combines complementary cues according to their reliability rather than treating all evidence as equally trustworthy [13]. Third, biological robustness is commonly achieved through regulated responses and functional fallback under perturbation, although such safeguards involve trade-offs rather than universal performance gains [14].

Within robot calibration, a prominent bio-inspired route uses evolutionary or swarm processes for parameter search and hybrid model optimization [20,21]. SGIS-RC follows a different but complementary route. Its inspiration lies in inference-time regulation: nuisance-selective physical estimation, complementary global–local discrepancy learning, and support-dependent attenuation of the learned response. These are engineering abstractions of biological information-processing principles, not claims of physiological equivalence.

### 2.2. Range-Constrained Calibration and Identifiability

Distance-constrained calibration replaces full pose observations with geometric invariants. Ball-bar and distance-sphere procedures recover informative combinations of kinematic errors with modest instrumentation [7,8], while cable and range-sensor methods extend this principle to automated and single-sensor acquisition [9,10,11]. The recent single draw-wire formulation of Boschetti and Sinico is particularly close in measurement economy, but it relies on deliberately selected configurations that isolate individual parameters under its prescribed geometry [11]. More generally, robot-calibration observability depends on the parameter convention, gauge, measurement geometry, and excitation [2,3,22]. Singular-spectrum truncation is a classical response to ill-conditioned inverse problems [23]; SGIS-RC applies it only after removing the local tangent generated by the anchor and bias nuisance variables.

### 2.3. Hybrid Compensation and Deployment Validity

Data-driven calibration increasingly complements geometric identification with learned models for effects that are difficult to parameterize explicitly [16]. Representative recent approaches combine kinetostatic modeling with Gaussian-process residual compensation [17], select informative calibration poses through Bayesian experimental design [18], and reduce measurement demand through active-learning hybrid calibration [19]. These methods demonstrate the value of combining physical structure with flexible statistical models, but their measurement assumptions and deployment domains differ from the unknown-anchor scalar-range setting considered here.

The present residual model uses low-order polynomial structure for global trends and a Matérn–Nyström basis for local variation [24,25,26]. Leverage and extrapolation diagnostics can indicate when a query is weakly supported by the fitted design, but they are not calibrated predictive probabilities [27,28]. SGIS-RC therefore treats support control as part of the prediction rule: support decay attenuates the local Nyström contribution, or the complete residual under the alternative joint-residual route, while leverage and amplitude controls bound the learned response. The methodological distinction is not a claim of distribution-shift robustness; it is an explicit rule governing how learned compensation is permitted to act as data support weakens.

## 3. Materials and Methods

### 3.1. Problem Formulation and Measurement Regimes

#### 3.1.1. Single-Range Observation

For a six-axis serial robot, the standard DH transform of joint *j* is [29]

(1) Tjj−1(θ)=Rotz(qj+θj)Transz(dj)Transx(aj)Rotx(αj),

and $p\left(q;\theta \right)\in R^{3}$ denotes the end-point translation. The nominal model is $\theta_{0}$, with correction Δθ. Four conventional gauge directions ($\Delta d_{1}$, $\Delta \alpha_{6}$, $\Delta \theta_{1}$, and $\Delta \theta_{6}$) are fixed; the remaining 20 DH coordinates are represented in type-normalized units and constrained by physically conservative bounds summarized in Section 3.3.2.

For observation *i*,

(2) $$y_{i}={∥p(q_{i};\theta_{0}+\Delta \theta )-a∥}_{2}+b+r\left(q_{i}\right)+\epsilon_{i},$$

where $a\in R^{3}$ is the anchor, *b* is an additive range bias, r(q) collects unresolved configuration-dependent effects, and $\epsilon_{i}$ is measurement noise. Only $\left(q_{i},y_{i}\right)$ are used for estimation; the auxiliary ABB XYZ fields are excluded because they are not independently validated Cartesian ground truth.

#### 3.1.2. Measurement Contracts

Table 1 summarizes the unknown- and fixed-anchor measurement contracts.

The U-track is the primary setting. After an initial robust nuisance fit, let $\kappa_{n}$ be the condition number of the scaled anchor–bias Jacobian and $u_{b}=\left|b-b_{c}\right|/h_{b}$ the normalized utilization of the bias interval. To avoid fitting a numerically unsupported bias,

(3) $$\mathrm{freeze}\left(b\right)=I\;\left[\kappa_{n}>10^{3}\;\vee \;u_{b}\geq 0.995\right].$$

If the condition holds, *b* is set to zero and the anchor is re-estimated. The L-track instead fixes the anchor (configured for ABB; derived from training-only nominal kinematics for HSR-C) and uses it unchanged across methods in the same split. The two tracks are analyzed separately because neither the nuisance state nor the supported physical interpretation is identical.

Figure 1 summarizes the measurement geometry and the two contracts.

### 3.2. SGIS-RC

#### 3.2.1. Bio-Inspired Design Abstraction

SGIS-RC is bio-inspired at the level of regulatory organization. The nuisance-projected physical stage implements selective filtering by removing anchor–bias variation before determining which kinematic directions are permitted to adapt. The unified residual field combines smooth global and local kernel components to represent complementary adaptation scales. Leverage, support, and amplitude controls then regulate the learned correction according to the numerical and configuration-space support available at deployment. Together, these correspondences provide a principle-level organization of the estimator while leaving its mathematical components and estimand unchanged. All fitted quantities remain robot-kinematic or statistical variables, and no physiological equivalence or biological necessity is implied. SGIS-RC is therefore positioned as a bio-inspired data-driven engineering framework rather than a biological model.

#### 3.2.2. Nuisance-Projected Physical Spectrum

Let $J_{\theta }$ and $J_{n}$ denote the scaled Jacobians of the geometric range with respect to the active kinematic and nuisance coordinates. Before scaling,

(4) $$\frac{\partial {\hat{y}}_{\mathrm{phys}}}{\partial a}=\frac{a-p}{{∥p-a∥}_{2}},\;\frac{\partial {\hat{y}}_{\mathrm{phys}}}{\partial b}=1.$$

At iteration *t*, robust Huber weights $W_{t}$ are obtained from the current physical residuals. If $Q_{n}$ spans $W_{t}^{1/2}J_{n}$, the kinematic sensitivity orthogonal to the nuisance tangent is

(5) $${\tilde{J}}_{t}=\frac{\left(I-Q_{n}Q_{n}^{⊤}\right)W_{t}^{1/2}J_{\theta }}{\sqrt{\sum_{i}W_{t,ii}}}=U\Sigma V^{⊤}.$$

Only directions satisfying $\sigma_{j}/\sigma_{1}\geq 10^{-3}$ and $\sigma_{1}/\sigma_{j}\leq 10^{4}$ are admissible. If $r_{E}$ is the smallest dimension containing 97% of the squared singular-value energy and $r_{\mathrm{eff}}$ the admissible numerical rank,

(6) $$k=\min\{\max\left(r_{E},1\right),r_{\mathrm{eff}},6\}.$$

Thus, the physical stage operates in a compact predictive subspace rather than treating all DH coordinates as independently identifiable.

#### 3.2.3. Robust Constrained Physical Estimation

Let $z\in R^{k}$ be the retained spectral coordinates, *u* the active nuisance increment, and $\xi ={\left[z^{⊤},u^{⊤}\right]}^{⊤}$. Each local update solves the bounded M-estimation problem

(7) $$\begin{array}{c} {\hat{\xi }}_{t}=\underset{\xi \in B_{t}}{\arg\;\min}\sum_{m}\rho_{s_{t}}\;\left(\Psi_{t,m}\left(\xi \right)\right),\\ \Psi_{t}={\left[r_{\mathrm{range}}^{⊤},\;\sqrt{\lambda_{\Sigma }}{\left(D_{\Sigma }z\right)}^{⊤},\;\sqrt{\lambda_{n}}u^{⊤},\;\sqrt{\lambda_{p}}e_{\mathrm{box}}^{⊤},\;\sqrt{\lambda_{g}}\mathrm{vec}{\left(p-p_{0}\right)}^{⊤}/s_{g}\right]}^{⊤}. \end{array}$$

where $\rho_{s_{t}}$ is the soft-$L_{1}$ loss and $D_{\Sigma }$ increases regularization along weaker retained directions. The feasible set and geometric trust constraints restrict the update to physically conservative departures from the nominal model. The residual-learning target is then the intact physical discrepancy

(8) $$t_{i}=y_{i}-{\hat{y}}_{\mathrm{phys},i}.$$

No orthogonality constraint is imposed on $t_{i}$; any remaining physical–residual overlap is therefore measurable rather than hidden by construction.

#### 3.2.4. Unified Global–Local Residual Field

Joint coordinates are normalized from the current fit set. To avoid polynomial blow-up outside the training box, each normalized coordinate *x* uses the rational $C^{1}$ extension

$$c\left(x\right)=\left\{\begin{array}{cc} x, & |x|\leq 1,\\ \mathrm{sgn}\left(x\right)\;\left(1+\frac{e}{1+e/m}\right), & |x|>1, \end{array}\right.\;e=\left|x\right|-1,\;m=0.25.$$

The global basis $\phi_{g}$ contains an intercept, fourth-order univariate Chebyshev terms, and low-order pairwise interactions. Local structure is represented by an anisotropic Matérn-3/2 kernel,

(9) $$k\left(x,x^{\prime }\right)=\left(1+\sqrt{3}d_{\ell }\right)\exp\left(-\sqrt{3}d_{\ell }\right),\;d_{\ell }^{2}=\sum_{j}\frac{{\left(x_{j}-x_{j}^{\prime }\right)}^{2}}{\ell_{j}^{2}},$$

and a deterministic Nyström map based on at most 256 farthest-point landmarks *Z*,

(10) $$\phi_{\ell }\left(x\right)=K_{xZ}{\left(K_{ZZ}+\varepsilon I\right)}^{-1/2}.$$

The two bases are concatenated once, $\phi =[\phi_{g},\phi_{\ell }]$, and estimated jointly by Huber block-ridge regression,

(11) $$\hat{\beta }=\underset{\beta }{\arg\;\min}\left\{\frac{1}{n}\sum_{i\in F}w_{i}\rho_{H}\;\left(t_{i}-\phi {\left(q_{i}\right)}^{⊤}\beta \right)+\beta^{⊤}\Lambda \beta \right\}.$$

This single objective preserves complementary global and local structure without model switching.

#### 3.2.5. Support-Controlled Deployment

For the final design matrix Φ and residual-fit weights $W_{r}$, define the robust scale operator

$$S\left(z\right)=\max\;\left\{0.05\;\mathrm{mm},\;1.4826\;{\mathrm{median}}_{i}\left|z_{i}-{\mathrm{median}}_{j}z_{j}\right|\right\}.$$

With physical residual target $t_{i}=y_{i}-{\hat{y}}_{\mathrm{phys},i}$ and post-fit residual $e_{i}=t_{i}-\phi {\left(q_{i}\right)}^{⊤}\hat{\beta }$, let $\sigma_{\mathrm{phys}}=S\left(t\right)$ and $\sigma_{\mathrm{fit}}=S\left(e\right)$. Then

(12) $$G=\Phi^{⊤}W_{r}\Phi +n\Lambda ,\;P=G^{+},\;h\left(q\right)=\phi {\left(q\right)}^{⊤}P\phi \left(q\right),\;\rho \left(q\right)=\frac{\sigma_{\mathrm{phys}}^{2}}{\sigma_{\mathrm{phys}}^{2}+\sigma_{\mathrm{fit}}^{2}h\left(q\right)}.$$

The factor ρ(q) is a deterministic shrinkage score; it is not interpreted as a calibrated probability. Let d(q) be the ARD-scaled distance from the training joint-space box, normalized by the median positive training-neighbor scale. A flat-core Wendland envelope gives

(13) $$s\left(q\right)=\left\{\begin{array}{cc} 1, & d(q)\leq 8,\\ {\left(1-\tau \right)}^{4}\left(1+4\tau \right), & 8<d(q)<64,\\ 0, & d(q)\geq 64, \end{array}\right.\;\tau =\frac{d(q)-8}{56}.$$

Let $\sigma_{t}=S\left(t\right)$, $R=\max\{\max_{i}|t_{i}|,\sigma_{t}\}$, and $B=R+4\sigma_{t}$. The smooth amplitude map is

$$A\left(v\right)=\left\{\begin{array}{cc} v, & |v|\leq R,\\ \mathrm{sgn}\left(v\right)\;\left[R+\frac{(B-R)e}{(B-R)+e}\right], & |v|>R, \end{array}\right.\;e=\left|v\right|-R,$$

so that it is exactly identity over the fitted residual range and approaches the finite bound *B* smoothly outside it. For a deployment route m∈{joint,local}, the predictor is written as

(14) $${\hat{y}}_{m}\left(q\right)={\hat{y}}_{\mathrm{phys}}\left(q\right)+r_{m}\left(q\right),\;m\in \left\{\mathrm{joint},\mathrm{local}\right\},$$

where the route-specific corrections $r_{\mathrm{joint}}$ and $r_{\mathrm{local}}$ are defined in Section 3.2.6. The leverage factor ρ(q) attenuates the learned correction as leverage increases, whereas the effect of the support envelope depends on the selected route. For the joint-residual route, s(q)=0 removes the complete residual correction; for the local-block route, it removes only the local residual component while retaining the amplitude-bounded global component. These controls regulate the magnitude and support of the learned correction but do not constitute a guarantee of low out-of-support prediction error.

#### 3.2.6. Support-Application Selection

The support rule is evaluated through two candidate deployment routes using the same fitted coefficients. With *g* and *ℓ* denoting the global Chebyshev and local Nyström components,

(15) $$\begin{array}{c} r_{\mathrm{joint}}\left(q\right)=\rho \left(q\right)s\left(q\right)A\;\left(g(q)+\ell (q)\right),\\ r_{\mathrm{local}}\left(q\right)=\rho \left(q\right)A\;\left(g(q)+s(q)\ell (q)\right). \end{array}$$

The joint-residual route suppresses both components when support decays, whereas the local-block route retains the amplitude-bounded global component and attenuates only the local kernel term. The route is selected on validation data by lexicographic MAE, CVaR_95_, and RMSE, with a $10^{-12}\;\mathrm{mm}$ numerical tie tolerance. Selection is frozen before the train–validation refit; test labels are never used for route choice, threshold choice, or error filtering. This formulation addresses a specific support-application failure mode without implying general distribution-shift robustness.

Figure 2 summarizes the physical, residual, and deployment stages.

#### 3.2.7. Selection and Refit

The retained dimension *k* and deployment route are selected using training and validation data only. With both decisions fixed, all fit-dependent quantities are re-estimated on the combined training and validation data. The held-out test partition is then evaluated once, without observation removal or test-driven model selection.

### 3.3. Evaluation Design

#### 3.3.1. Data and Partitions

Table 2 summarizes the two public-source workbooks. Joint angles are converted to radians and ranges remain in millimeters.

All protocols target a 70%/15%/15% train/validation/test allocation. Random holdouts characterize interpolation-oriented performance. Configuration-group partitions keep near-duplicate joint configurations in the same split, while spatial blocks withhold regions of normalized joint space. Ordered blocks withhold contiguous workbook rows and are interpreted only as a row-order correlation or drift proxy because timestamps are unavailable. Two partition sets serve complementary purposes. The first uses seeds {260,906, 365,635, 470,364, 575,093, 679,822} for protocol characterization, ablations, and mechanism analyses. Because the support-placement analysis on this set informed the final route specification, these results are interpreted as development and mechanism evidence rather than as the sole post-specification assessment. Performance after specification freeze is therefore evaluated separately on a second pre-specified partition set described below. Variation across either set is interpreted as within-workbook partition sensitivity rather than replication across independent robots.

#### 3.3.2. Comparators and Metrics

The U-track compares SGIS-RC with nominal and robust NLS, the nuisance-projected physical model, an otherwise matched conventional hybrid without deployment control, and four joint-only regressors: Extra Trees, histogram gradient boosting, kernel ridge regression, and a multilayer perceptron. Joint-only hyperparameters are selected on validation data and refitted on train+validation. The L-track compares nominal NLS, robust NLS, and SGIS-RC under a common fixed anchor and zero bias. Ablations remove the residual field, global basis, local basis, or leverage factor. Published evolutionary and particle-swarm calibration results are discussed but not numerically cross-ranked because their measurement contracts and implementations are not protocol matched.

MAE is the primary metric; RMSE, median error, upper quantiles, CVaR_95_ [32], and maximum error are retained for tail analysis. Values are mean ± sample standard deviation across repeated partitions and are interpreted as partition-sensitivity summaries rather than population-level significance tests. Additional analyses include a same-fit full $2^{3}=8$ support/leverage/amplitude factorial, training-derived quartile stratification of $q_{1}$–$q_{6}$ and observed range, and a validation-only one-at-a-time (OAT) sensitivity analysis covering 15 factors and 31 declared settings. The factorial reports MAE, CVaR_95_ absolute error, and maximum absolute error; these analyses characterize model behavior and are not used for held-out test-set model selection.

All fits use deterministic double precision. The complete fixed numerical settings used in the reported evaluation are listed in Table 3.

#### 3.3.3. Pre-Specified Post-Specification Outer-Partition Evaluation

Before any outcomes from the second partition set were generated or inspected, the SGIS-RC architecture, candidate route set, numerical settings, route-selection rule, metric definitions, dataset identities, and outer-partition seed list were fixed. The second seed set is {860,906, 965,635, 170,364, 275,093, 379,822} and is disjoint from that used in the mechanism and ablation analyses.

For each dataset and protocol, the outer-test partition is assigned first. The remaining development rows are then separated into training and validation partitions under the same protocol semantics. The identifiable dimension *k* is determined from training data only; the joint-residual versus local-block route is selected on validation data by the fixed MAE–CVaR_95_–RMSE lexicographic rule; the selected *k* and route are then held fixed while a single final model is refitted on training plus validation and evaluated once on the outer test. The same frozen specification is used across all pre-specified outer partitions.

Both partition sets draw from the same two public workbooks. The second set therefore provides a post-specification assessment of partition-level stability, but it is not an external validation study and does not represent previously unseen robot measurements.

## 4. Results

Unless otherwise stated, the following protocol, ablation, and mechanism analyses report the first partition set; the separately pre-specified post-specification outer-partition evaluation is reported in Section 4.6.

### 4.1. Unknown-Anchor Performance Across Partition Protocols

Table 4 summarizes the U-track results on the first partition set; Table 5 gives the joint-only comparison and Figure 3 extends the view to the two block partitions.

SGIS-RC remains below 0.24mm and 0.06mm MAE in the ABB and HSR-C configuration-group cells, respectively, with the conventional hybrid yielding slightly lower mean MAE in both configuration-group cells. These results support a structured and auditable interpretation of SGIS-RC, in which the physical subspace, residual components, and deployment controls remain explicit while predictive performance remains competitive in the evaluated in-support protocols. The repeated-partition variability reflects within-workbook split sensitivity rather than independent-robot uncertainty.

Among the joint-only models, kernel ridge regression is the strongest competitor. It is slightly lower than SGIS-RC on HSR-C (0.0488 versus 0.0506mm), whereas SGIS-RC remains substantially lower on ABB (0.2372 versus 0.4680mm). The other three regressors are less accurate on the configuration-group partitions. The comparison therefore highlights a cost–benefit trade-off: simpler joint-only prediction can be competitive, while SGIS-RC retains explicit measurement geometry, physical/residual attribution, and deployment control.

### 4.2. Support-Shift Stress and Tail Risk

The complete stress-protocol summaries are given in Table 6; the SGIS-RC rows appear as the block portions of Figure 3.

The block results reveal a dataset-dependent trade-off. SGIS-RC is competitive on ABB spatial blocks and substantially better on ABB ordered blocks, whereas the conventional hybrid remains better on HSR-C spatial blocks and slightly better on HSR-C ordered blocks. The same-fit comparison in Figure 4 isolates the support-placement mechanism: attenuating the complete residual can suppress a useful global component together with the local correction, whereas local-block attenuation preserves the global term. Because this mechanism analysis informed the final route set, it is interpreted as design evidence rather than as a stand-alone confirmation of final predictive performance. The pre-specified post-specification outer-partition evaluation in Section 4.6 provides the complementary assessment after specification freeze. Taken together, the results show that support control changes failure behavior without guaranteeing extrapolation or distribution-shift robustness.

### 4.3. Fixed-Anchor Paired Evidence

The fixed-anchor results in Table 7 and Figure 5 isolate residual compensation from anchor estimation.

Against robust NLS, the paired MAE improvement is 1.53–1.64mm on ABB and 11.19–11.44mm on HSR-C across the random and configuration-group protocols of the first partition set. Under this simplified nuisance contract, the result isolates the contribution of the adaptive residual branch. These values characterize the fixed-anchor contract only and should not be transferred to the unknown-anchor estimand.

### 4.4. Identifiable Spectrum and Component Ablation

Table 8 gives the component ablations, while Figure 6 and Figure 7 show the fitted spectrum and paired effects for the principal removals.

Both datasets retain k=3 physical directions under configuration grouping, despite larger nuisance-projected numerical ranks (14 for ABB and 12 for HSR-C). Removing the residual field raises MAE from 0.237mm to 2.272mm on ABB and from 0.051mm to 11.895mm on HSR-C, showing that the physical branch is a structured attribution and reference component rather than the dominant accuracy source. The global and local branches contribute differently by dataset, while removing leverage changes configuration-group MAE by less than 0.001mm; this is consistent with a deployment-regulation role rather than an interpolation-accuracy claim.

### 4.5. Deployment-Control, Sensitivity, and Variable-Stratified Analysis

The deployment-control analysis applies support (*S*), leverage (*L*), and amplitude (*A*) switches in all $2^{3}=8$ combinations while reusing identical fitted coefficients and held-out rows within each split. Table 9 reports the resulting MAE, CVaR_95_ absolute error, and maximum absolute error. Effects are negligible under configuration grouping but become larger under block holdouts. On ABB spatial blocks, for example, S1/L0/A0 reduces the mean triplet from 0.827/3.728/4.649 mm for S1/L1/A1 to 0.665/3.161/3.918 mm. On HSR-C spatial blocks, amplitude-off states reduce the corresponding values from 0.277/1.356/1.917 mm to approximately 0.262/1.124/1.493 mm. The controls therefore regulate the learned correction but do not impose a monotonic improvement in mean or tail error. Support-route placement is analyzed separately in Figure 4, and global/local residual contributions are quantified in Table 8.

Figure 8 and Table 10 summarize the one-at-a-time sensitivity analysis. Global regularization is the most responsive tested setting: increasing it from $10^{-5}$ to $3\times 10^{-5}$ changes the five-partition HSR-C spatial validation MAE from 1.224849 to 1.553763 mm, an absolute shift of 0.328914 mm. Local regularization is the next most responsive factor (0.055517 mm on ABB spatial blocks), while all remaining factors produce maximum absolute changes of at most 0.015884 mm over the tested levels. The analysis therefore identifies a localized sensitivity of the residual regularization under spatial holdout rather than uniform insensitivity of the numerical controls. The sensitivity study is based only on validation errors and does not alter the reported test configuration.

To examine variable-wise error heterogeneity, Table 11 stratifies held-out scalar-range MAE by training-derived quartiles of each joint coordinate and observed range for the configuration-group and spatial-block protocols. All held-out rows are retained; “–” denotes an empty training-derived stratum rather than a removed test row. The grouped partitions are comparatively uniform, whereas the spatial blocks expose substantially larger variable-dependent error heterogeneity, consistent with the support-shift results in Table 6.

### 4.6. Post-Specification Outer-Partition Evaluation

A second pre-specified partition set evaluates the frozen SGIS-RC specification across the same two workbooks. Five partitions are used for each dataset and protocol, giving 40 SGIS-RC outer evaluations under one common model specification. Table 12 summarizes the results. Configuration-group MAE is 0.2306±0.0148 mm for ABB and 0.0493±0.0021 mm for HSR-C. Random-partition errors remain comparable, whereas the block protocols are more variable: ABB spatial and ordered MAEs are 1.355±0.568 and 2.005±0.531 mm, while HSR-C spatial and ordered MAEs are 0.177±0.038 and 0.050±0.007 mm.

The validation selector chooses the local-block route in 38 of 40 partitions and the joint-residual route in two ABB ordered partitions. The retained physical dimension is k=3 in 37 partitions, k=4 in one ABB spatial partition, and k=2 in one HSR-C spatial and one HSR-C ordered partition. Matched configuration-group comparisons preserve the ordering observed in the first partition set: the conventional hybrid gives 0.2244±0.0151 mm versus SGIS-RC 0.2306±0.0148 mm on ABB and 0.0478±0.0015 mm versus 0.0493±0.0021 mm on HSR-C. On HSR-C spatial blocks, the hybrid also remains lower (0.138±0.021 versus 0.177±0.038 mm). In contrast, the hybrid is numerically unstable on the pre-specified ABB spatial partitions, including one divergent fit; this event is retained in the evaluation record and is not used to infer superiority of SGIS-RC. Overall, the second partition set shows similar in-support scalar-range accuracy to the first partition set while preserving the dataset- and protocol-dependent character of block-shift behavior.

## 5. Discussion

### 5.1. Bio-Inspired Methodological Interpretation

The results support the proposed regulatory organization while keeping the biological interpretation at a principle level. Nuisance projection implements selective filtering by restricting physical adaptation to directions that remain distinguishable after anchor–bias variation is removed. The large physics-only error, particularly on HSR-C, shows that this branch should be interpreted as a structured physical reference rather than as the principal source of predictive accuracy; most of the scalar-range compensation is supplied by the learned residual field. The global-only and local-only ablations further reveal dataset-dependent residual structure, supporting their complementary use within a common regularized model.

The support-route analysis provides a mechanism-level interpretation of reliability-dependent adaptation. Applying support attenuation to the complete residual can suppress a useful smooth global component together with the local correction, whereas local-block attenuation confines support decay to the local component. This mechanism analysis informed the final route set and is therefore interpreted primarily as design evidence. The separately pre-specified outer-partition evaluation provides a complementary post-specification assessment: configuration-group errors remain low in both workbooks, while the block protocols preserve dataset-dependent behavior, including a lower HSR-C spatial mean for the conventional hybrid. Because both partition sets reuse the same workbooks, the comparison strengthens partition-level stability evidence without establishing external data-level independence.

Support, leverage, and amplitude are consequently best interpreted as deterministic applicability controls rather than calibrated uncertainty measures or guarantees of distribution-shift robustness [27,28]. The full $2^{3}=8$ same-fit results in Table 9 show that their effects are non-monotonic under block holdouts: disabling a control can reduce a particular mean or tail error. The OAT analysis likewise identifies a marked HSR-C spatial sensitivity to global regularization. These observations favor an auditable deployment interpretation in which the controls shape how correction is applied, rather than a claim that they uniformly improve predictive or tail performance.

The biomimetic contribution is consequently methodological rather than physiological. Selective filtering, complementary adaptation scales, and reliability-dependent attenuation provide an engineering organization for established statistical and numerical tools. SGIS-RC does not claim biological necessity or physiological equivalence; instead, its contribution lies in making the physical, residual, and deployment roles explicit and independently auditable. This also distinguishes SGIS-RC from evolutionary or swarm-based calibration approaches [20,21], where the nature-inspired element primarily enters through the optimization strategy. For pure scalar-range prediction, simpler models can remain competitive—kernel ridge regression, for example, attains a slightly lower mean MAE on HSR-C—so the principal benefit of SGIS-RC is the structured and auditable organization of measurement geometry, residual compensation, and deployment control.

### 5.2. Limitations and Outlook

The present evidence is based on two public robot workbooks rather than independent acquisition campaigns or a population of robot systems. The second pre-specified partition set avoids using the original partition assignments as the only post-specification assessment, but it draws from the same underlying observations. The results therefore characterize within-workbook post-specification partition stability rather than transfer to new robot units, sensors, or acquisition conditions. The retained spectral coordinates should likewise be interpreted as locally supported predictive directions under the adopted measurement contract, not as verified recovery of the complete DH parameter vector. The block protocols, variable-stratified results, deployment-control factorial, sensitivity analysis, and post-specification outer evaluation characterize complementary aspects of support dependence under the available data.

Future work should extend the evaluation to independent robot experiments, external Cartesian metrology, and protocol-matched reproductions of representative hybrid or nature-inspired calibration methods. Such studies would provide the additional evidence required to connect the present scalar-range compensation framework to broader calibration and deployment settings.

## 6. Conclusions

Under the post-specification outer-partition evaluation, SGIS-RC achieves configuration-group MAEs of 0.2306±0.0148 mm on ABB IRB120 and 0.0493±0.0021 mm on HSR-C JR680. The matched conventional hybrid remains slightly lower in these two cells (0.2244±0.0151 mm and 0.0478±0.0015 mm), indicating that the value of SGIS-RC lies not in uniform mean-error superiority, but in combining numerically supported physical adaptation with explicit residual and deployment structure. The broader stress, sensitivity, and factorial analyses further show that block-shift behavior and deployment-control effects are dataset and protocol dependent, while preserving a clear separation between predictive compensation and unsupported physical-parameter interpretation. Taken together, these results support an auditable formulation in which identifiable physical structure, flexible discrepancy modeling, and deployment regulation play distinct but complementary roles. This separation provides a principled basis for future measurement-compensation and calibration systems operating under limited, nuisance-coupled, or weakly informative sensing conditions.

## Figures and Tables

**Figure 1:**
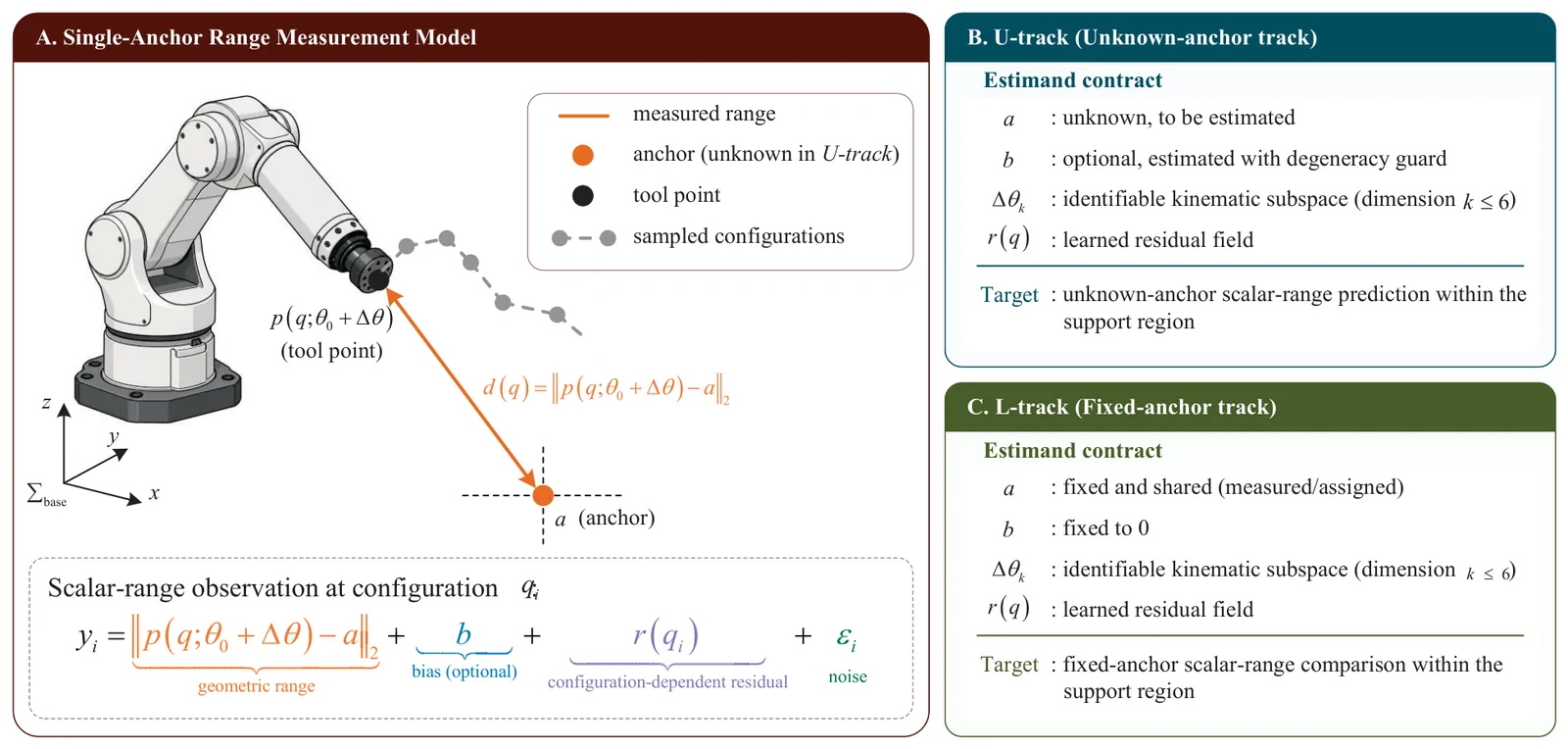
Single-anchor range observation and the unknown- and fixed-anchor measurement contracts.

**Figure 2:**
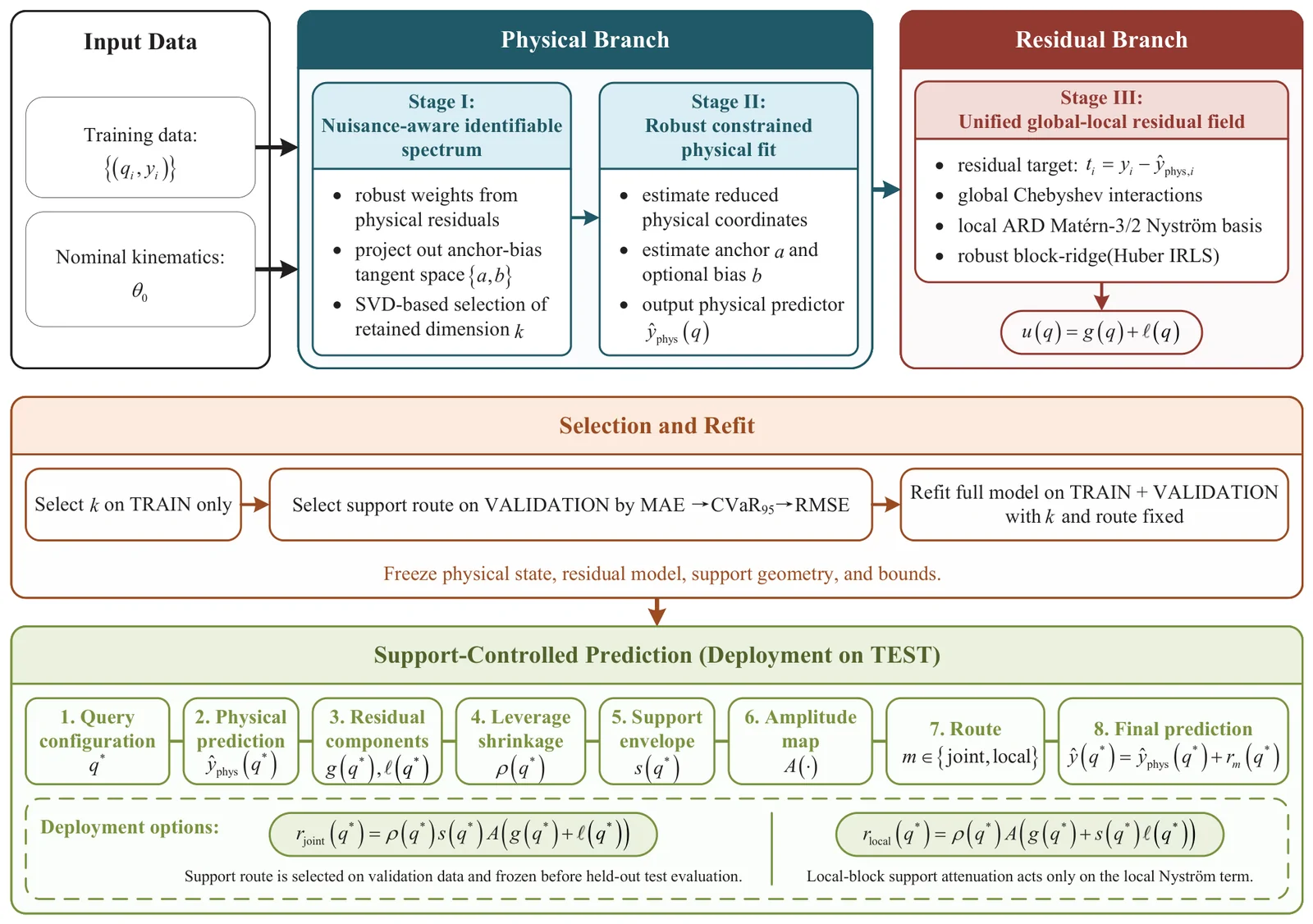
SGIS-RC estimation chain. At the level of bio-inspired design abstraction, nuisance projection provides selective filtering, the unified residual field supplies complementary global–local adaptation, and deployment control regulates the learned correction when support is weak. Here, $q^{*}$ denotes a query joint configuration at deployment; the superscript ∗ is not a statistical-significance marker.

**Figure 3:**
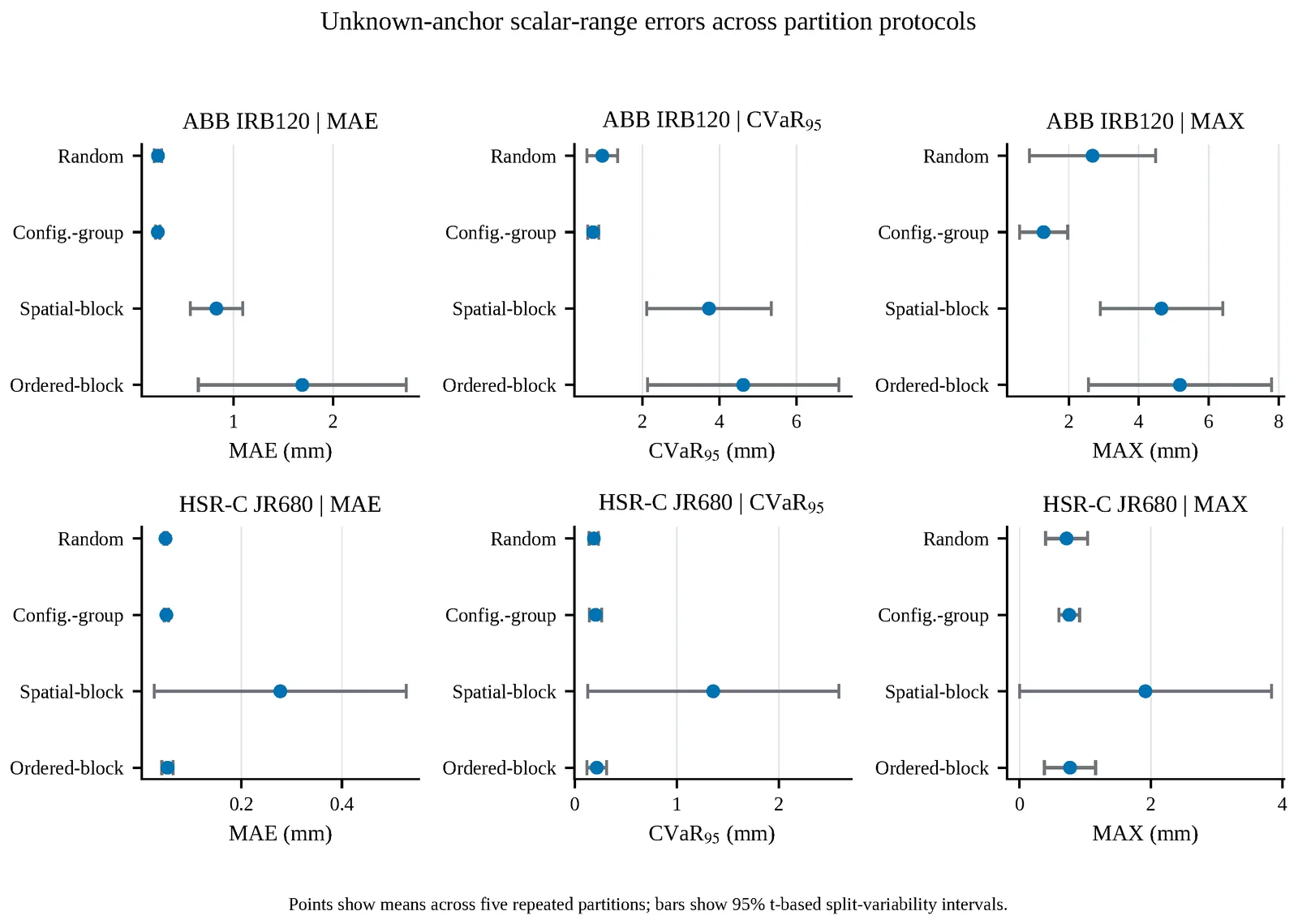
SGIS-RC unknown-anchor scalar-range errors across the four partition protocols on the first partition set. Points show means across five repeated partitions and bars show 95% Student-*t* intervals describing split variability. Random and configuration-group partitions represent the principal in-support protocol types within this set; spatial blocks withhold regions of normalized joint space, and ordered blocks withhold contiguous workbook rows as a row-order correlation or drift proxy.

**Figure 4:**
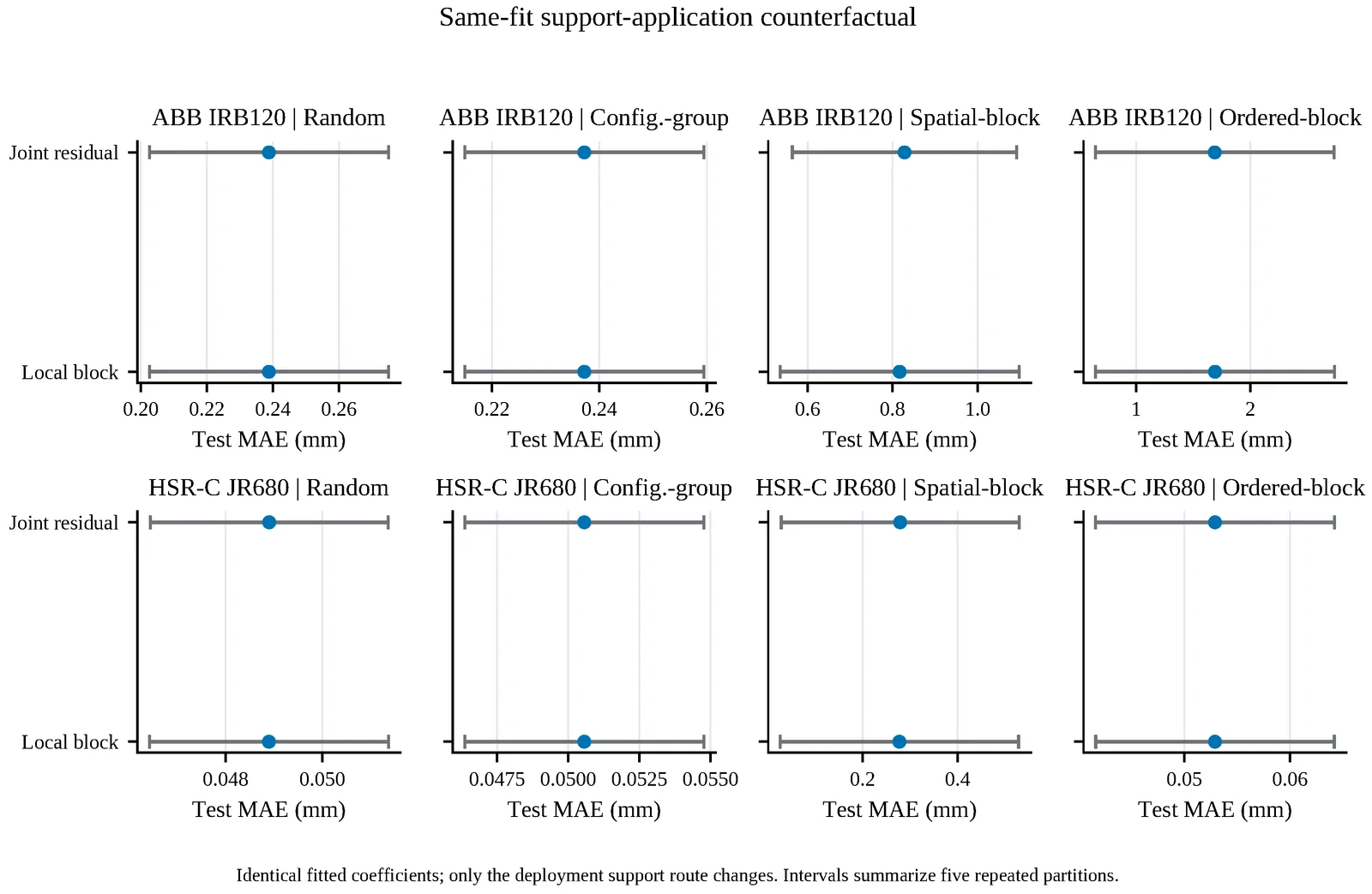
Same-fit comparison of the joint-residual and local-block deployment policies. The two routes reuse identical fitted physical parameters, residual coefficients, held-out rows, and error metrics, so the comparison isolates support placement alone. Horizontal scales are panel specific.

**Figure 5:**
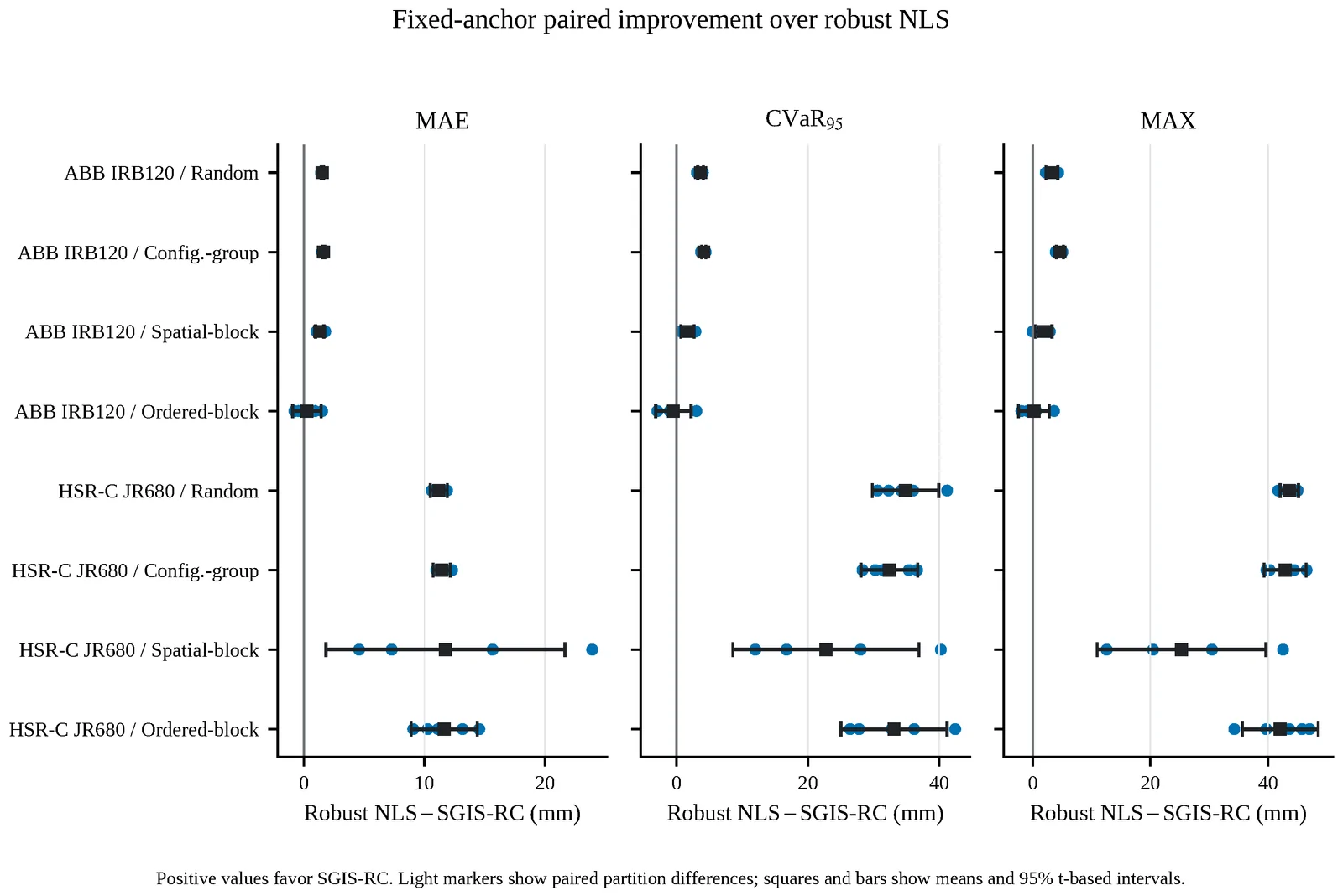
L-track paired improvement over robust NLS using common test rows, anchor, zero-bias specification, and partitions. Light markers show paired partition differences; squares indicate their means, and bars show 95% Student-*t* intervals. Positive differences favor SGIS-RC.

**Figure 6:**
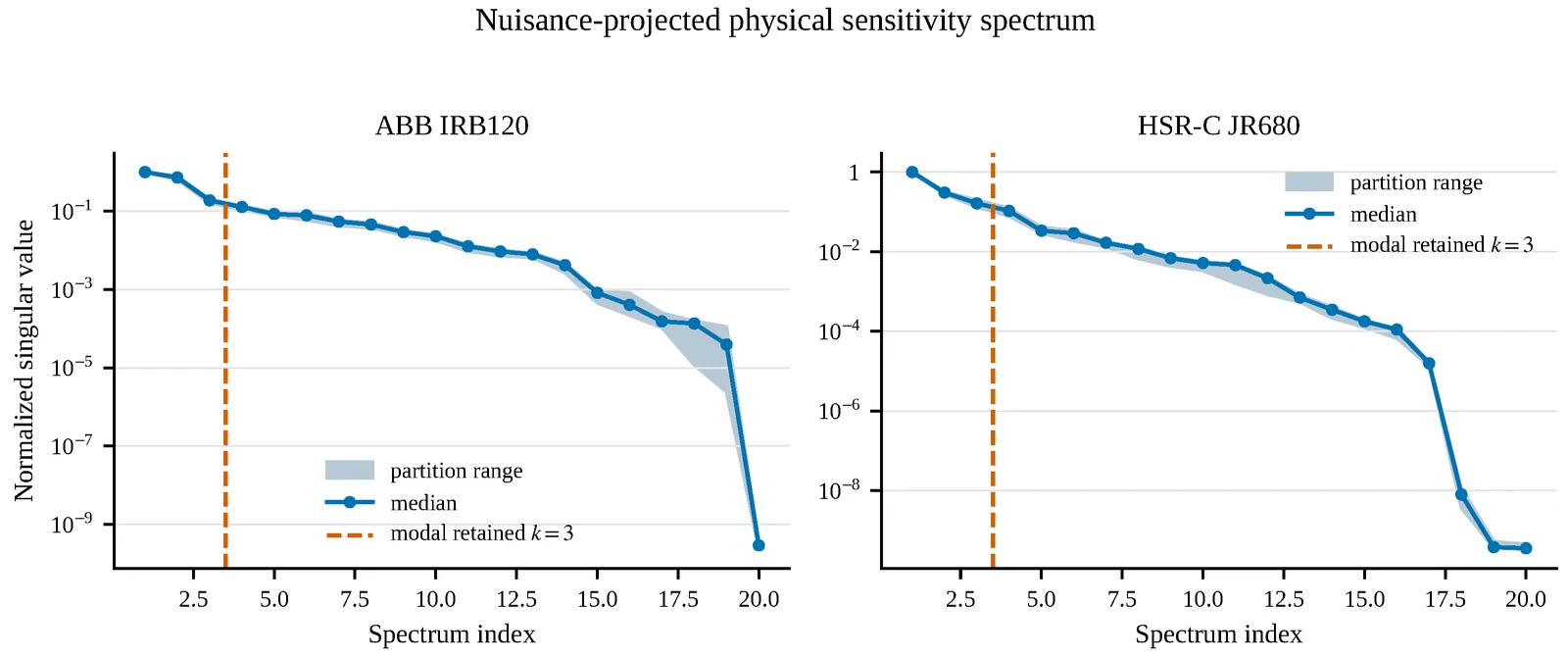
Nuisance-projected singular spectra across the repeated training partitions. The shaded range is descriptive partition variability; the retained dimensions are predictive numerical choices under the adopted scaling and nuisance projection rather than globally identifiable DH parameters.

**Figure 7:**
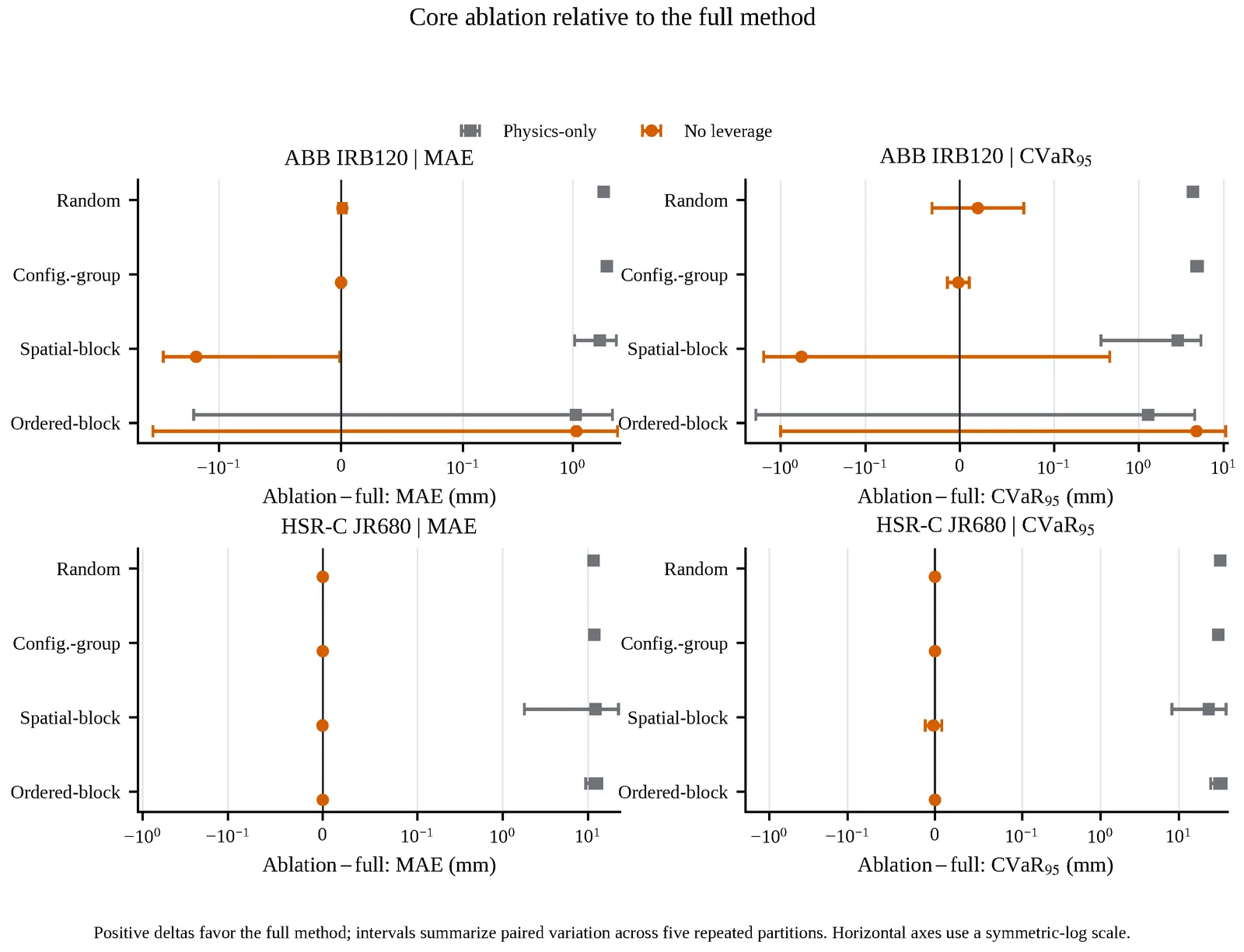
Paired U-track effects of removing the complete residual field (physics-only) or the leverage factor across the four partition protocols. Points show mean ablation-minus-full differences over five repeated partitions; bars summarize paired split variability. Horizontal axes use a symmetric-log scale to display small signed changes and large positive ablation effects on the same panels. Global-only and local-only variants are reported in Table 8.

**Figure 8:**
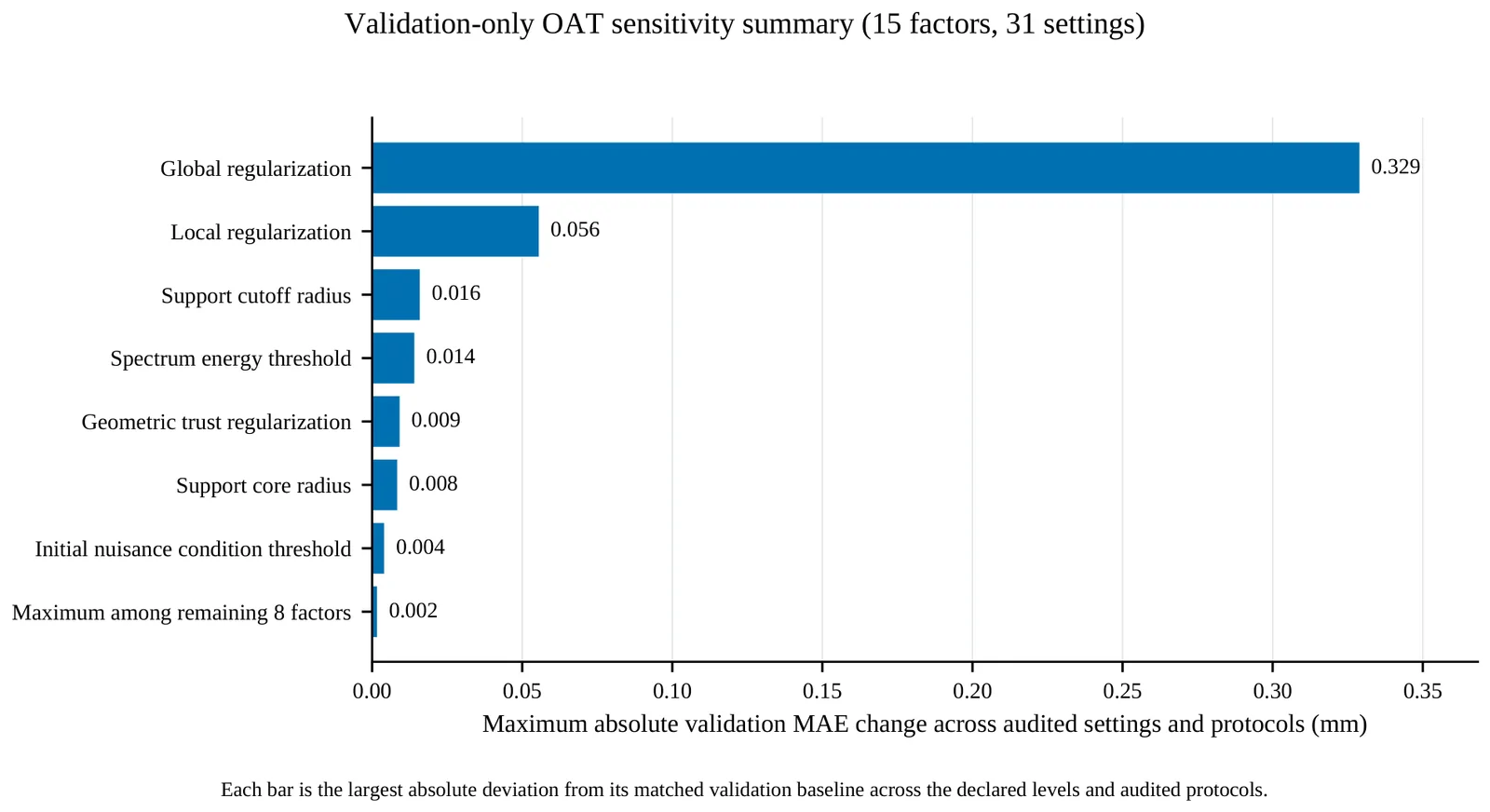
One-at-a-time validation sensitivity. Each bar reports the maximum absolute MAE deviation from its matched validation baseline across the declared levels and protocols. The most responsive factors are shown individually; the final bar gives the maximum among the remaining eight factors. Test labels are not used to select or retune numerical settings.

**Table 1:** Unknown- and fixed-anchor measurement contracts.

Track	Nuisance Specification	Estimand
U-track	Anchor estimated jointly; bias retained unless its nuisance geometry is degenerate	Unknown-anchor scalar-range prediction
L-track	Anchor fixed and shared within each split; b=0	Fixed-anchor scalar-range comparison

**Table 2:** Real-data sources used in the study.

Dataset	Rows	Retained Variables	Source
ABB IRB120	1042	$q_{1}\ldots q_{6}$, *L*; auxiliary XYZ excluded	RobotCali [30]
HSR-C JR680	2017	$q_{1}\ldots q_{6}$, *L*	HSR-RobotCali [31]

**Table 3:** Complete numerical settings used in the SGIS-RC evaluation. Values are fixed engineering and numerical controls rather than test-set-optimized constants.

Group	Fixed Values
Physical bounds	Length/offset coordinate scale: 5 mm with ±20 mm bounds; twist/joint-offset scale: 0.5° with ±2.3° bounds; anchor scale: 50 mm; bias scale: 10 mm; initial anchor margin: 2500 mm; initial bias limit: ±300 mm
Nuisance guard	Initial nuisance evaluations 1200; initial scaled condition threshold $10^{3}$; boundary-freeze ratio 0.995; three nuisance multistarts with 0.20 perturbation fraction
Spectrum	Relative threshold $10^{-3}$; maximum condition $10^{4}$; energy target 0.97; retained dimension 1–6; rank tolerance $10^{-10}$
Physical optimization	Spectral regularization 0.01; nuisance regularization $10^{-4}$; parameter-bound penalty 100; geometric-trust regularization 10; RMS trust scale 5 mm; maximum RMS shift 2 mm; normalized step bound 4
Solver	Outer iterations 20; local function evaluations per iteration 180; local $\mathrm{ftol}=\mathrm{xtol}=\mathrm{gtol}=10^{-10}$; minimum line-search scale 1/8192; relative objective tolerance $10^{-8}$; normalized-step tolerance $10^{-3}$; relative optimality tolerance $10^{-5}$
Residual fit	Chebyshev degree 4 with cross-degree 2; Nyström components 256; ARD length scales learned from at most 384 fit samples with bounds [0.05,20]; global/local ridge $10^{-5}$; kernel jitter $10^{-8}$; Huber δ=1.345; IRLS iterations 500; tolerance $10^{-8}$; relaxation 0.5
Deployment	Support distance normalized by the median positive training-neighbor scale; core radius 8; cutoff radius 64; amplitude margin scale 4; rational-$C^{1}$ coordinate-extension margin 0.25
Route selection	Joint-residual versus local-block support placement; validation-only lexicographic MAE–CVaR_95_–RMSE selection; $10^{-12}$ mm numerical tie tolerance

**Table 4:** U-track held-out scalar-range errors (mm) on the first partition set, reported as mean ± sample standard deviation over five repeated splits. The conventional hybrid uses the same physical fit and an unconstrained residual field.

Dataset	Protocol	SGIS-RC MAE	Conv. Hybrid MAE	Robust NLS MAE	SGIS-RC CVaR_95_	SGIS-RC MAX
ABB	Random	0.239±0.029	0.234±0.023	1.308±0.037	0.955±0.321	2.681±1.454
ABB	Configuration-group	0.237±0.018	0.228±0.015	1.320±0.029	0.718±0.115	1.282±0.554
HSR-C	Random	0.049±0.002	0.048±0.002	8.402±0.487	0.185±0.037	0.715±0.256
HSR-C	Configuration-group	0.051±0.003	0.049±0.003	8.285±0.509	0.203±0.048	0.756±0.127

**Table 5:** Configuration-group U-track MAE (mm) for the four joint-only families and SGIS-RC. Each family is selected using validation data and refitted on training plus validation; no method is selected from the held-out results. Entries are mean ± sample standard deviation over five splits.

Method	ABB IRB120	HSR-C JR680
SGIS-RC	0.237±0.018	0.051±0.003
Extra Trees	4.272±0.470	0.094±0.011
Histogram gradient boosting	4.264±0.223	0.876±0.115
Kernel ridge regression	0.468±0.030	0.049±0.003
Multilayer perceptron	3.572±0.519	0.396±0.070

**Table 6:** U-track stress protocols (mm), comparing SGIS-RC with the conventional unconstrained hybrid. Entries are mean ± sample standard deviation over the same five splits.

Dataset	Protocol	Method	MAE	CVaR_95_	MAX
ABB	Spatial-block	SGIS-RC	0.827±0.213	3.728±1.304	4.649±1.410
		Conv. hybrid	0.784±0.164	4.423±1.003	5.188±1.119
ABB	Ordered-block	SGIS-RC	1.691±0.843	4.619±2.003	5.177±2.108
		Conv. hybrid	3.297±2.365	20.228±20.411	20.873±20.806
HSR-C	Spatial-block	SGIS-RC	0.277±0.202	1.356±0.992	1.917±1.545
		Conv. hybrid	0.189±0.104	0.784±0.268	1.054±0.306
HSR-C	Ordered-block	SGIS-RC	0.053±0.009	0.214±0.077	0.768±0.314
		Conv. hybrid	0.051±0.008	0.197±0.070	0.768±0.312

**Table 7:** L-track fixed-anchor MAE (mm), reported as mean ± sample standard deviation over five repeated splits. Paired robust-NLS-minus-SGIS-RC differences are given in the text; positive differences favor SGIS-RC.

Dataset	Protocol	Nominal	Robust NLS	SGIS-RC
ABB	Random	3.089±0.107	1.769±0.065	0.239±0.026
ABB	Configuration-group	3.197±0.192	1.872±0.087	0.236±0.018
HSR-C	Random	11.755±0.588	11.241±0.572	0.049±0.002
HSR-C	Configuration-group	12.011±0.593	11.489±0.573	0.051±0.003

**Table 8:** Configuration-group component ablations. Entries are mean ± sample standard deviation of held-out scalar-range errors (mm) over five repeated splits.

Dataset	Variant	MAE	CVaR_95_	MAX
ABB	Full	0.237±0.018	0.718±0.115	1.282±0.554
ABB	Physics-only	2.272±0.126	5.591±0.479	6.578±0.719
ABB	Global-only	0.417±0.032	2.844±0.352	3.685±0.354
ABB	Local-only	0.267±0.020	0.953±0.136	1.727±0.749
ABB	No leverage	0.237±0.017	0.717±0.114	1.289±0.516
HSR-C	Full	0.051±0.003	0.203±0.048	0.756±0.127
HSR-C	Physics-only	11.895±0.486	31.877±2.042	41.902±1.646
HSR-C	Global-only	0.051±0.003	0.203±0.048	0.757±0.127
HSR-C	Local-only	1.629±0.347	8.524±1.020	12.973±2.175
HSR-C	No leverage	0.051±0.003	0.203±0.048	0.756±0.127

**Table 9:** Same-fit full $2^{3}=8$ deployment-control results. Each numeric cell reports MAE/ CVaR_95_/ MAX (mm), averaged over five repeated partitions. *S*, *L*, and *A* denote support, leverage, and amplitude control, respectively (0 = off; 1 = on). All eight states reuse identical fitted coefficients and held-out rows within each split; no state is refitted or used for policy selection.

Control State	Random	Configuration-Group	Spatial-Block	Ordered-Block
ABB IRB120				
S0/L0/A0	0.240/0.974/2.858	0.237/0.717/1.289	0.756/3.920/4.671	3.191/13.848/14.510
S0/L0/A1	0.240/0.974/2.858	0.237/0.717/1.289	0.755/3.905/4.630	2.766/9.393/10.246
S0/L1/A0	0.239/0.955/2.681	0.237/0.718/1.282	0.816/3.603/4.493	1.695/4.643/5.210
S0/L1/A1	0.239/0.955/2.681	0.237/0.718/1.282	0.817/3.607/4.505	1.691/4.619/5.177
S1/L0/A0	0.240/0.974/2.858	0.237/0.717/1.289	0.665/3.161/3.918	3.190/13.835/14.497
S1/L0/A1	0.240/0.974/2.858	0.237/0.717/1.289	0.665/3.161/3.918	2.766/9.392/10.245
S1/L1/A0	0.239/0.955/2.681	0.237/0.718/1.282	0.826/3.726/4.644	1.695/4.643/5.210
**S1/L1/A1**	0.239/0.955/2.681	0.237/0.718/1.282	0.827/3.728/4.649	1.691/4.619/5.177
HSR-C JR680				
S0/L0/A0	0.049/0.185/0.716	0.051/0.203/0.756	0.261/1.122/1.489	0.053/0.214/0.768
S0/L0/A1	0.049/0.185/0.716	0.051/0.203/0.756	0.276/1.354/1.913	0.053/0.214/0.768
S0/L1/A0	0.049/0.185/0.716	0.051/0.203/0.756	0.261/1.124/1.493	0.053/0.214/0.768
S0/L1/A1	0.049/0.185/0.716	0.051/0.203/0.756	0.277/1.355/1.917	0.053/0.214/0.768
S1/L0/A0	0.049/0.185/0.715	0.051/0.203/0.756	0.262/1.123/1.489	0.053/0.214/0.768
S1/L0/A1	0.049/0.185/0.715	0.051/0.203/0.756	0.277/1.354/1.913	0.053/0.214/0.768
S1/L1/A0	0.049/0.185/0.715	0.051/0.203/0.756	0.262/1.124/1.493	0.053/0.214/0.768
**S1/L1/A1**	0.049/0.185/0.715	0.051/0.203/0.756	0.277/1.356/1.917	0.053/0.214/0.768

Bold control-state labels identify the complete SGIS-RC deployment-control setting (S1/L1/A1), not the best-performing result.

**Table 10:** Numerical levels used in the OAT sensitivity analysis and the largest absolute validation-MAE response for each factor. Support radii are in normalized ARD-distance units; regularization and penalty values refer to the internally scaled numerical parameterization.

Factor	Baseline	Low	High	Max. $|\Delta {\mathrm{MAE}}_{\mathrm{val}}|$ (mm)	Location of Maximum
Spectrum relative threshold	$1\times 10^{-3}$	$3\times 10^{-4}$	$3\times 10^{-3}$	0.000000	–
Spectrum maximum condition	$1\times 10^{4}$	$3\times 10^{3}$	$3\times 10^{4}$	0.000000	–
Spectrum energy threshold	0.97	0.95	0.99	0.014063	HSR-C spatial, high
Spectrum maximum dimension	6	4	8	0.000000	–
Initial nuisance condition	1000	300	3000	0.003984	HSR-C spatial, low
Nuisance boundary-freeze ratio	0.995	0.980	0.999	0.000000	–
Spectral regularization	0.01	0.003	0.03	0.000002	HSR-C spatial, high
Nuisance regularization	$1\times 10^{-4}$	$3\times 10^{-5}$	$3\times 10^{-4}$	0.000002	HSR-C spatial, high
Parameter-bound penalty	100	30	300	0.000000	–
Geometric-trust regularization	10	3	30	0.009170	ABB spatial, low
Global regularization	$1\times 10^{-5}$	$3\times 10^{-6}$	$3\times 10^{-5}$	0.328914	HSR-C spatial, high
Local regularization	$1\times 10^{-5}$	$3\times 10^{-6}$	$3\times 10^{-5}$	0.055517	ABB spatial, low
Support core radius	8	4	16	0.008350	HSR-C spatial, high
Support cutoff radius	64	32	128	0.015884	HSR-C spatial, high
Output-bound margin scale	4	2	8	0.001635	HSR-C spatial, high

**Table 11:** Held-out scalar-range MAE (mm) stratified by training-derived quartiles of $q_{1}$–$q_{6}$ and observed range. Q1–Q4 denote the quartiles; “–” denotes an empty training-derived stratum.

Dataset	Protocol	Variable	Q1	Q2	Q3	Q4
ABB IRB120	Configuration-group	$q_{1}$	0.244	0.233	0.251	0.229
		$q_{2}$	0.222	0.234	0.217	0.277
		$q_{3}$	0.236	0.235	0.241	0.233
		$q_{4}$	0.230	0.223	0.232	0.255
		$q_{5}$	0.228	0.245	0.235	0.243
		$q_{6}$	0.251	0.215	0.241	0.240
		Observed range	0.269	0.215	0.222	0.238
ABB IRB120	Spatial-block	$q_{1}$	1.168	0.769	0.609	0.708
		$q_{2}$	0.434	0.614	0.941	1.144
		$q_{3}$	1.297	0.945	0.324	0.611
		$q_{4}$	0.499	0.490	0.493	1.538
		$q_{5}$	0.593	0.346	1.248	1.301
		$q_{6}$	1.451	0.755	0.414	0.656
		Observed range	1.295	0.784	0.480	0.447
HSR-C JR680	Configuration-group	$q_{1}$	–	0.061	0.048	0.042
		$q_{2}$	0.039	0.046	0.047	0.065
		$q_{3}$	0.064	0.053	0.047	0.040
		$q_{4}$	0.053	0.054	0.039	0.061
		$q_{5}$	0.066	0.048	0.043	0.053
		$q_{6}$	0.052	0.061	0.064	0.041
		Observed range	0.049	0.044	0.057	0.053
HSR-C JR680	Spatial-block	$q_{1}$	–	0.342	0.158	0.033
		$q_{2}$	0.302	0.058	0.232	0.383
		$q_{3}$	0.416	0.261	0.142	0.231
		$q_{4}$	0.330	0.194	0.041	0.472
		$q_{5}$	0.275	0.059	0.375	0.401
		$q_{6}$	0.302	0.368	0.216	–
		Observed range	0.178	0.311	0.100	0.478

**Table 12:** Post-specification outer-partition evaluation of the frozen SGIS-RC specification. Values are mean ± sample standard deviation over five pre-specified partition assignments on each public workbook. CVaR_95_ and MAX are absolute-error metrics in millimeters. Route and *k* columns report validation-selected counts.

Dataset	Protocol	MAE (mm)	CVaR_95_ (mm)	MAX (mm)	Route Counts	*k* Counts
ABB	Random	0.239±0.009	0.824±0.173	1.889±1.145	Local 5/5	3: 5/5
ABB	Configuration-group	0.231±0.015	0.716±0.111	1.440±0.923	Local 5/5	3: 5/5
ABB	Spatial-block	1.355±0.568	4.422±1.288	5.389±1.730	Local 5/5	3: 4/5; 4: 1/5
ABB	Ordered-block	2.005±0.531	5.310±0.645	5.876±0.619	Local 3/5; Joint 2/5	3: 5/5
HSR-C	Random	0.049±0.001	0.171±0.022	0.619±0.265	Local 5/5	3: 5/5
HSR-C	Configuration-group	0.049±0.002	0.175±0.053	0.495±0.240	Local 5/5	3: 5/5
HSR-C	Spatial-block	0.177±0.038	0.877±0.181	1.121±0.179	Local 5/5	3: 4/5; 2: 1/5
HSR-C	Ordered-block	0.050±0.007	0.175±0.060	0.556±0.300	Local 5/5	3: 4/5; 2: 1/5

## Data Availability

The numerical settings and audit results required to interpret the reported experiments are included in the manuscript. The implementation, experimental configurations, machine-readable evaluation manifests, and generated audit tables will be released in a public repository upon publication. The ABB IRB120 and HSR-C JR680 workbooks were obtained from their cited public source repositories; raw workbook files are not redistributed here, and reuse remains subject to the source repositories’ terms.

## Abbreviations

The following abbreviations are used in this manuscript:

ARD	Automatic relevance determination
CVaR	Conditional value at risk
DH	Denavit–Hartenberg
IRLS	Iteratively reweighted least squares
MAE	Mean absolute error
NLS	Nonlinear least squares
RMSE	Root-mean-square error
SGIS-RC	Spectrum-Guided Identifiable Single-Anchor Range Compensation

## References

[1] Roth Z.S., Mooring B.W., Ravani B. (1987). An Overview of Robot Calibration. IEEE J. Robot. Autom..

[2] Hollerbach J.M., Wampler C.W. (1996). The Calibration Index and Taxonomy for Robot Kinematic Calibration Methods. Int. J. Robot. Res..

[3] Khalil W., Gautier M., Enguehard C. (1991). Identifiable Parameters and Optimum Configurations for Robots Calibration. Robotica.

[4] Borm J.H., Meng C.H. (1991). Determination of Optimal Measurement Configurations for Robot Calibration Based on Observability Measure. Int. J. Robot. Res..

[5] Nubiola A., Bonev I.A. (2013). Absolute Calibration of an ABB IRB 1600 Robot Using a Laser Tracker. Robot. Comput.-Integr. Manuf..

[6] Li Z., Li S., Luo X. (2021). An Overview of Calibration Technology of Industrial Robots. IEEE/CAA J. Autom. Sin..

[7] Nubiola A., Bonev I.A. (2014). Absolute Robot Calibration with a Single Telescoping Ballbar. Precis. Eng..

[8] Joubair A., Bonev I.A. (2015). Kinematic Calibration of a Six-Axis Serial Robot Using Distance and Sphere Constraints. Int. J. Adv. Manuf. Technol..

[9] Gao G., Li Y., Liu F., Han S. (2021). Kinematic Calibration of Industrial Robots Based on Distance Information Using a Hybrid Identification Method. Complexity.

[10] Kang Z., Wang L., Sun A., Xu S., Wang B. (2024). Two-Step Calibration of 6-DOF Industrial Robots by Grouping Kinematic Parameters Based on Distance Constraints. Measurement.

[11] Boschetti G., Sinico T. (2024). A Novel Step-by-Step Procedure for the Kinematic Calibration of Robots Using a Single Draw-Wire Encoder. Int. J. Adv. Manuf. Technol..

[12] Wark B., Lundstrom B.N., Fairhall A. (2007). Sensory Adaptation. Curr. Opin. Neurobiol..

[13] Ernst M.O., Banks M.S. (2002). Humans Integrate Visual and Haptic Information in a Statistically Optimal Fashion. Nature.

[14] Kitano H. (2004). Biological Robustness. Nat. Rev. Genet..

[15] Pfeifer R., Lungarella M., Iida F. (2007). Self-Organization, Embodiment, and Biologically Inspired Robotics. Science.

[16] Chen T., Yang W., Li S., Luo X. (2025). Data-Driven Calibration of Industrial Robots: A Comprehensive Survey. IEEE/CAA J. Autom. Sin..

[17] Zhou Y., Chen C.Y., Tang Y., Wan H., Luo J., Yang G., Zhang C. (2024). A Comprehensive On-Load Calibration Method for Industrial Robots Based on a Unified Kinetostatic Error Model and Gaussian Process Regression. IEEE Trans. Instrum. Meas..

[18] Daş E., Touma T., Burdick J.W. (2025). Bayesian Optimal Experimental Design for Robot Kinematic Calibration. Proceedings of the 2025 IEEE International Conference on Robotics and Automation (ICRA).

[19] Selingue M., Olabi A., Thiery S., Bearee R. (2026). Data-Efficient, Fast and Autonomous Hybrid Calibration for Robot Positioning Accuracy Enhancement Using Active Learning-Based Method. J. Intell. Robot. Syst..

[20] Chen T., Li S., Qiao Y., Luo X. (2024). A Robust and Efficient Ensemble of Diversified Evolutionary Computing Algorithms for Accurate Robot Calibration. IEEE Trans. Instrum. Meas..

[21] Wei P., Deng X., Chen X., Li Z., Gan J., Shu H. (2026). Hybrid Mapping Polynomial Neural Network Based on PSO Optimization for Robot Calibration. IEEE Trans. Instrum. Meas..

[22] Jiang Z., Huang M. (2021). Stable Calibrations of Six-DOF Serial Robots by Using Identification Models with Equalized Singular Values. Robotica.

[23] Hansen P.C. (1990). Truncated Singular Value Decomposition Solutions to Discrete Ill-Posed Problems with Ill-Determined Numerical Rank. SIAM J. Sci. Stat. Comput..

[24] Trefethen L.N. (2019). Approximation Theory and Approximation Practice.

[25] Rasmussen C.E., Williams C.K.I. (2006). Gaussian Processes for Machine Learning.

[26] Williams C.K.I., Seeger M. (2001). Using the Nyström Method to Speed Up Kernel Machines. Proceedings of the Advances in Neural Information Processing Systems.

[27] Hoaglin D.C., Welsch R.E. (1978). The Hat Matrix in Regression and ANOVA. Am. Stat..

[28] Bartley M.L., Hanks E.M., Schliep E.M., Soranno P.A., Wagner T. (2019). Identifying and Characterizing Extrapolation in Multivariate Response Data. PLoS ONE.

[29] Denavit J., Hartenberg R.S. (1955). A Kinematic Notation for Lower-Pair Mechanisms Based on Matrices. J. Appl. Mech..

[30] Li Z., Li S., Francis A., Luo X. (2022). A Novel Calibration System for Robot Arm via an Open Dataset and a Learning Perspective. IEEE Trans. Circuits Syst. II Express Briefs.

[31] YWYucas HSR-RobotCali: HSR-C JR680 Robot Calibration Workbook. GitHub repository, 2022. Commit d505ce4. https://github.com/YWYucas/HSR-RobotCali/tree/d505ce4.

[32] Rockafellar R.T., Uryasev S. (2000). Optimization of Conditional Value-at-Risk. J. Risk.

